# Intergenerational effects of overfeeding on aversive learning in zebrafish (*Danio rerio*)

**DOI:** 10.1002/ece3.9423

**Published:** 2022-10-17

**Authors:** Hamza Anwer, Dominic Mason, Susanne Zajitschek, Daniel Hesselson, Daniel W. A. Noble, Margaret J. Morris, Malgorzata Lagisz, Shinichi Nakagawa

**Affiliations:** ^1^ Evolution & Ecology Research Centre and School of Biological, Earth and Environmental Sciences University of New South Wales Sydney New South Wales Australia; ^2^ Diabetes and Metabolism Division Garvan Institute of Medical Research Darlinghurst, Sydney New South Wales Australia; ^3^ Liverpool John Moores University School of Biological and Environmental Sciences Liverpool UK; ^4^ Centenary Institute and Faculty of Medicine and Health University of Sydney Sydney New South Wales Australia; ^5^ Division of Ecology and Evolution, Research School of Biology The Australian National University Canberra Australian Capital Territory Australia; ^6^ Department of Pharmacology, School of Medical Sciences University of New South Wales Sydney New South Wales Australia

**Keywords:** behavior, cognition, intergenerational, obesogenic diet, repeatability, zebrafish

## Abstract

The obesity epidemic is concerning as obesity appears to negatively impact cognition and behavior. Furthermore, some studies suggest that this negative effect could be carried across generations from both mothers and fathers although evidence is not consistent. Here, we attempt to address how obesogenic diets in the parental generation (F0) can impact offspring's cognition and anxiety intergenerationally (F1) in a zebrafish model. We compare both mean trait values and their variances. Using a multifactorial design, we created a total of four groups: F1T (treatment mothers × treatment fathers); F1M (treatment mothers × control fathers); F1P (treatment fathers × control mothers); and F1C (control mothers × control fathers, F1C); and subjected them to anxiety tank tests and aversive learning assays. When both parents were exposed, offspring (F1T) displayed the poorest aversive learning, while offspring that only had one parent exposed (F1P and F1M) learnt the aversive learning task the best. Zebrafish in all groups displayed no statistically significant differences in anxiety‐associated behaviors. Males and females also performed similarly in both anxiety and aversive learning assays. While all F1 groups had similar levels of fasting blood glucose, variance in glucose levels were reduced in F1P and F1T indicating the importance of investigating heteroskedasticity between groups. Furthermore, anxiety behaviors of these two groups appeared to be less repeatable. To our knowledge, this is the first study to test the intergenerational effects of an obesogenic diet on zebrafish cognition. Our multifactorial design as well as repeated tests also allowed us to disentangle maternal and paternal effects (as well as combined effects) and accurately detect subtle information such as between‐individual variation.

## Graphical abstract

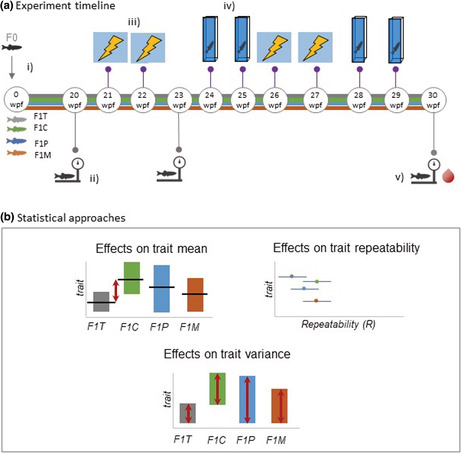

## 1 INTRODUCTION

The obesity epidemic is among the most serious and rapidly growing public health challenges of the 21st century (Seidell & Halberstadt, 2015). According to WHO, worldwide obesity has nearly tripled in the last 40–50 years (World Health Organization, 2017). This rapid increase is concerning because obesity is associated with a cluster of risk factors for cardiovascular disease and diabetes (i.e., insulin resistance, hyperglycemia, and hypertension; known as the ‘Metabolic Syndrome’; Alberti et al., 2009). This complex association of risk factors has been known for decades. However, recent research has highlighted a specific link between obesity and cognitive function (Smith et al., 2011). There is strong evidence for cognitive impairment in individuals with obesity (Elias et al., 2003; Prickett et al., 2015; Smith et al., 2011). It is also known that increased body mass index (BMI) and greater intake of unhealthy foods, high in fat, is associated with deficits in learning, memory, and executive functioning (Buie et al., 2019; Cordner & Tamashiro, 2015; Cournot et al., 2006).

Animal models are often used in experiments using obesogenic diets to investigate the underpinnings of detrimental effects on animal cognition (Castanon et al., 2015; Dickinson, 2012). As cognition involves processes such as learning and memory, measures of animal cognition usually revolve around learning paradigms which assess an animal's response to external stimuli (Shettleworth, 2001). One form of typical learning paradigms in animal studies is classical conditioning (Pavlov, 2010). Classical conditioning using an aversive stimulus, or hereafter, aversive conditioning, can be used to assess learning about danger cues (Shechner et al., 2014). Aversive conditioning has been used in several diet‐induced obesity studies in rodents to explore impacts on cognitive abilities (Reichelt et al., 2015; Yamada‐Goto et al., 2012). More recently, however, zebrafish (*Danio rerio*) have emerged as a valuable alternative in such studies (Macrì et al., 2020; Meguro et al., 2019).

Zebrafish are an excellent model in which to study the impacts of metabolic disorders as they possess similar pathophysiological pathways as mammals (Oka et al., 2010; Schlegel & Stainier, 2006; Zang et al., 2018). Zebrafish also have sophisticated sensory and motor systems, making them capable of learning in a variety of paradigms (Blaser & Vira, 2014; Pather & Gerlai, 2009; Sison & Gerlai, 2010; Spence et al., 2008). For instance, a recent study by Picolo et al. (2021) revealed how a high‐fat diet impacted memory as well as aggression and anxiety‐like behavior in zebrafish. However, no studies have explicitly tested the intergenerational effects of obesogenic diets on cognition in zebrafish. The zebrafish is also a promising animal model in both obesogenic diet studies (Zang et al., 2018) and behavioral neuroscience (Aoki et al., 2015). Zebrafish are cheap, reproduce in large numbers in short intervals, are easy to experimentally manipulate and possess a rich behavioral repertoire (Kalueff et al., 2013; Nguyen et al., 2013). Of relevance to this study, zebrafish are an external fertilizing species, which allows a more straightforward decoupling of maternal and paternal effects because the role of maternal responses in mediating effects is limited (Crean & Bonduriansky, 2014).

The maternal and paternal effects of diet‐induced obesity on offspring cognition have been investigated before in animal models (Basatemur et al., 2013; Yeung et al., 2017). For instance, it has been shown that high‐fat diet (HFD) consumption in mothers and fathers impairs learning and memory in both rat and mouse offspring (Hasebe et al., 2021; Lin et al., 2021; Zhou et al., 2018). Although, studies rarely examine the combined effects of maternal and paternal exposure, usually focusing on one or the other independently. This represents an opportunity to address a gap in the literature. It is important to note that maternal and paternal effects are viewed as key elements in generating phenotypic variation in offspring (Bonduriansky & Day, 2009; Bonduriansky & Head, 2007; Nettle & Bateson, 2015; Puy et al., 2021). Therefore, nutritional stress in the parental generation could generate phenotypic variation in the offspring generation (Luca et al., 2010). In our modern obesogenic environment, high‐caloric foods are in abundance and readily available in comparison to the past (Lev‐Ran, 2001; Vandevijvere et al., 2015). Indeed, we can see such abundant availability of food as “an evolutionarily novel stressor,” requiring organisms to develop an adaptive response to the obesogenic environment (Tsatsoulis et al., 2013). Yet, it remains largely unresolved whether intergenerational transmission (i.e., parental effects) allows for adaptive evolution in the face of novel environmental stress (i.e., an obesogenic diet; O'Dea et al., 2016; Uller, 2008).

Here, we address the question of how obesogenic diets in the parental generation (F0) can impact offspring cognition intergenerationally (F1) in zebrafish (*Danio rerio*). We conducted a multifactorial experiment by breeding four F1 groups from F0 fish from our previous work (Anwer, O'Dea, et al., 2022) with and without exposure to an obesogenic diet (i.e., treatment mothers × treatment fathers, or F1T; treatment mothers × control fathers, or F1M; treatment fathers × control mothers, or F1P; control mothers × control fathers, F1C). In our previous study (Anwer, O'Dea, et al., 2022), we examined effects of an obesogenic diet on the immediate generation (F0). In this study, offspring groups of the F0 generation (F1) are subjected to aversive learning assays to answer our main question—“What is the effect of an obesogenic parental diet on offspring cognition in terms of its magnitudes and variability?” (i.e., changes in means and variances between groups). Variance analysis is commonly neglected in studies in favor of mean differences. However, as individuals vary in their level of cognitive performance, repeated trials are necessary to assess individual consistency. Here, analyzing components of variance such as repeatability becomes necessary as well as crucial to understanding how parental effects impact individuals. Notably, our multifactorial design allows us to investigate treatment effects, as well as discern maternal and paternal influences. We predict that both maternal and paternal exposures to an obesogenic diet will result in poorer learning responses and may generate more variation, which would be greater in offspring where both parents were exposed. Also, we conduct novel tank tests for anxiety, a behavioral measure that has been shown to be closely associated with cognitive processes (Darcet et al., 2014). In addition, we examine sex differences, an important biological variable in experiments (Zajitschek et al., 2020), although we do not have a priori predictions on the direction of these differences. Finally, we explore the effects of parental diet on commonly explored parameters such as offspring body weight and fasting blood glucose, which we expect to be adversely affected with higher body weight in offspring of exposed parents as well as higher levels of fasting blood glucose, as shown in previous zebrafish work (although maternal and paternal effects in these studies were not separated; do Carmo Rodrigues Virote et al., 2020; Türkoğlu et al., 2021).

## 2 MATERIALS AND METHODS

### 2.1 Experimental subjects and design

#### 2.1.1 Zebrafish Husbandry

We raised and maintained Mixed Wildtype (WT) zebrafish stock in a Tecniplast Zebtec System at 28°C under a 12‐h light:12‐h dark cycle at the Garvan Institute of Medical Research, Sydney, Australia. The wild‐type stock was derived from of a mixture of Tübingen long fin, AB and other unidentified strains (which had been interbred for 8–10 generations to increase genetic diversity). Adult zebrafish were housed in 3.5L tanks (max 24 fish per 3.5‐litre tank), and larval zebrafish until 1 month of age in 1.1L tanks (max 50 larval zebrafish per 1.1L tank). All tanks received recirculating water (pH 7–8, conductivity 500–2500 μs). We fed zebrafish larvae a standard facility diet of *Paramecium* twice daily up until 10–12 dpf, at which point they were weaned onto live *Artemia* (twice a day) and dried fish food (once a day). The Garvan Animal Ethics Committee approved all animal experimental procedures described here (approval: ARA 18_18), with handling and maintenance following established protocols.

#### 2.1.2 Parental diets

At 12 weeks postfertilization (wpf), adult parental zebrafish (F0) were assigned to either obesogenic (overfeeding) or control diets (see Appendix S1 for details on how the F0 generation was produced). Diets were adapted from Oka et al. (2010) and were a method of overfeeding due to its simplicity in producing an obese phenotype (Zang et al., 2018). The diet consisted of freshly hatched Artemia, dried decapsulated Artemia (INVE Artemia Shell Free: An Artemia Nauplii Alternative), and commercially available fish food (O.range GROW‐L). We fed both groups Artemia twice daily (the first feed freshly hatched artemia and the second feed dried artemia): zebrafish in the obesogenic group received 60 mg/fish/day (i.e., 1440 mg/tank equating to 720 mg per feed), while zebrafish in the control group received 5 mg/fish/day (i.e., 120 mg/tank equating to 60 mg per feed). We provided all obesogenic and control tanks with 200 mg of fish food once in the morning to assist with macronutrient requirements. Diets were maintained for 18 weeks at which point the F1 generation was produced.

#### 2.1.3 Experimental overview

**FIGURE 1:**
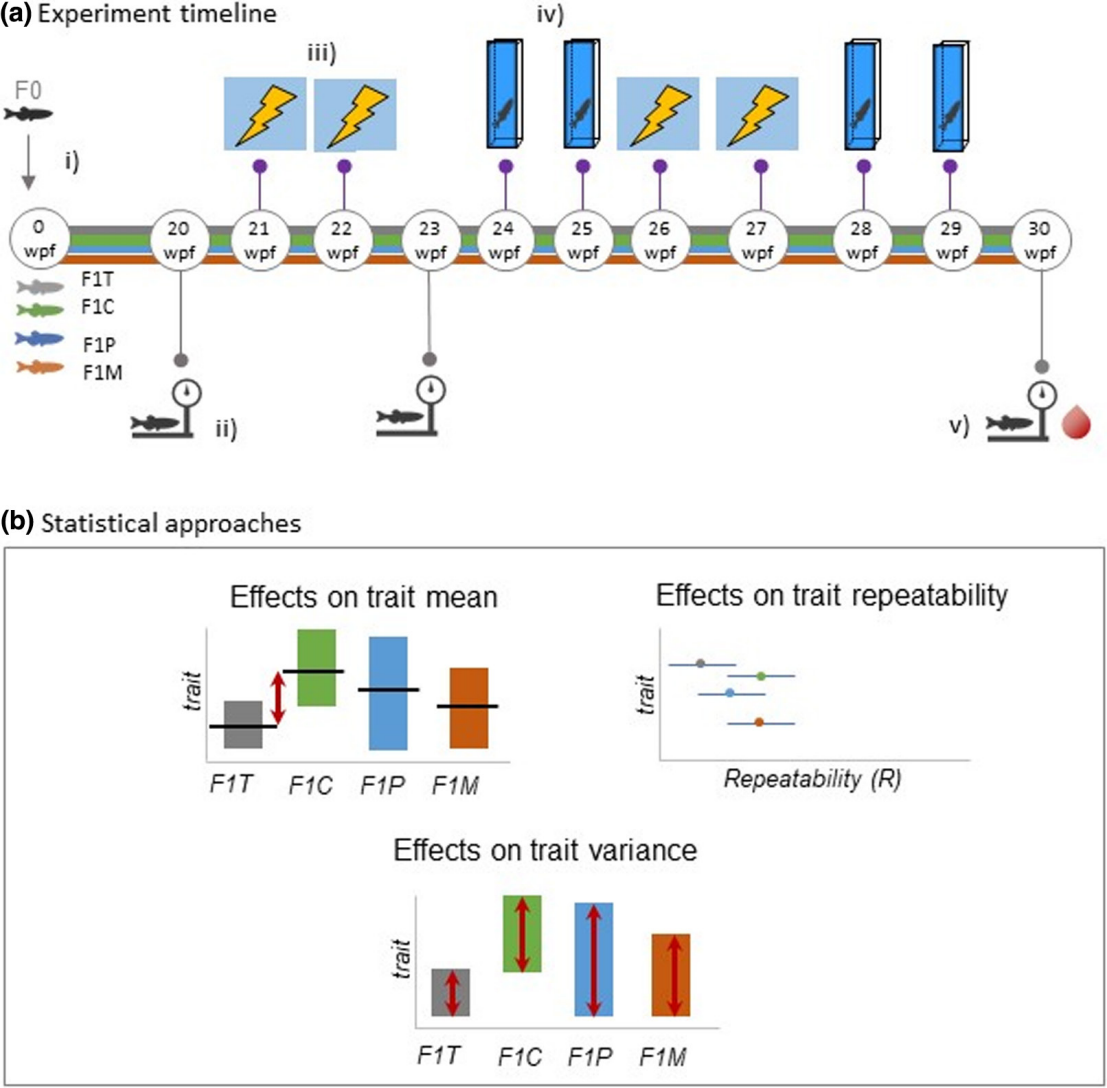
Experimental overview and statistical approaches. (a) Experimental overview and timeline: (i) At 12 weeks postfertilization (wpf), parental zebrafish (F0) were assigned to either obesogenic (overfeeding) or control diets; zebrafish in the obesogenic group received 60 mg/fish/day (i.e., 1440 mg/tank equating to 720 mg per feed), while zebrafish in the control group received 5 mg/fish/day (i.e., 120 mg/tank equating to 60mg per feed); Diets were maintained for 18 weeks at which point the F1 generation was produced. F0 control and treatment groups bred to produce 4 groups of F1 generation fish: F1T (treatment mothers × treatment fathers); F1C (control mothers × control fathers, F1C; F1P (treatment fathers × control mothers); F1M (treatment mothers × control fathers); (ii) Weighing zebrafish begins at 20 wpf (first of 3 measurements); (iii) F1 zebrafish subjected to aversive learning experiments at 21 wpf and 22 wpf (first 2 of 4 trials) (iv) F1 zebrafish first subjected to anxiety tank tests at 24 wpf and 25 wpf (first 2 of 4 trials); (v) final body weight measurements and all fish are sacrificed for fasting blood glucose measurements; (b) Statistical approaches used to analyze data: mean and variance differences calculated between groups through the use of mixed models and; repeatability of behavior estimates calculated as the proportion of between‐group (between‐individual) variance out of total variance.

We produced the F1 generation by breeding parental zebrafish within control and treatment groups (i.e., males and females were selected from the same group and not interbred between groups); and also created sex‐specific crosses between parental control and treatment groups. This design allowed us to investigate treatment effects, as well as discern maternal and paternal influences. We created a total of four groups: F1T (treatment mothers × treatment fathers); F1M (treatment mothers × control fathers); F1P (treatment fathers × control mothers); and F1C (control mothers × control fathers, F1C). We balanced sex ratio and family representation within each group for statistical independence. F1 fish were void of any diet manipulation and fed a standard facility diet. The experimental protocol began with F1 zebrafish aged at 20 wpf, at which point we took our first weight measurement. From 21 wpf, we subjected F1 zebrafish to aversive learning and anxiety tests (Figure 1). Multiple tests were required to obtain repeatability estimates (see Section 4). Due to competitive hierarchies in relation to food access among zebrafish in tanks (Paull et al., 2010), we used 20 fish from each tank per group (*n* = 40 F1T, *n* = 40 F1M; *n* = 40 F1P; *n* = 40 F1C; total 160; Figure 1) excluding 2 of the heaviest males and 2 of the heaviest females from F1C (likely 4 most dominant individuals), and 2 of the lightest males and 2 of the lightest females from F1T, F1P, and F1M (likely 4 most subordinate individuals). Zebrafish that died during the experiment were replaced with a counterpart from a spare tank (each group had an allocated spare tank). We weighed zebrafish a total of three times (at 20 wpf, 23 wpf and 30 wpf) before culling them for fasting blood glucose measurements at the end of the experiment (Figure 1).

## 3 BEHAVIORAL ASSAYS AND OTHER MEASUREMENTS

### 3.1 Aversive learning assay

We used an aversive conditioning assay to investigate learning ability in offspring of zebrafish fed obese and control diets. Behavioral tests were performed and filmed using the Zantiks [AD] fully automated behavioral testing boxes (Zantiks Ltd., Cambridge, UK) following the protocol described by Mason et al. (2021) (also see Appendix S2). We quantified learning as the difference in time spent in the conditioned stimulus (CS+) (in this case, a visual cue associated with an aversive jolt), before and after the aversive experience (difference = time spent in the CS+ during baseline ‐ time spent in the CS+ during probe). A higher difference indicates less time spent in the CS+ following the aversive experience. Differences are expressed as seconds per minute. Each fish experienced the aversive learning assay a total of four times (sessions were separated by approximately 1–3 weeks; see Figure 1).

### 3.2 Anxiety assay

We followed the procedure as described in Anwer et al. (2021), which involves filming zebrafish in a custom‐designed tank which has greater depth than traditionally used trapezoidal or cuboid tanks (traditional tanks typically range from ~15 to 20 cm, whereas custom‐designed tanks were 46 cm deep). Our work has shown that this type of tank generates more between‐individual differences and is suited for detecting subtle differences in behavior (Anwer et al., 2021). As described in our earlier work, we can measure several anxiety‐associated behaviors. However, since many of these behaviors are correlated, we focused our analysis on two highly repeatable, less correlated behaviors: (1) time spent in the low zone (seconds) and, (2) total distance travelled (cm). We subjected zebrafish to the anxiety assay a total of 4 times (the sessions were separated by approximately 1–3 weeks, see Figure 1). For each of the four assay sessions, we tested all fish in a single day. We pseudorandomized the order of fish being tested to account for the day of experiments, as well as the time of day. Trials began at 10 am and ended at 4 pm. We changed the water every hour (to ensure all fish were assayed in one day, we employed water changes on an hourly basis rather than a trial‐by‐trial basis) to minimize drops in temperature (water was maintained at ~28°C) and the effects of stress hormones from fish already trialed (Fontana et al., 2021; Pavlidis et al., 2013).

### 3.3 Body weight and fasting blood glucose

Body weight (g) measurements for F1 were taken at 20, 23, and 30 wpf using an AND EJ‐123 scale. A small case filled with water was placed on the scale and its weight was tared before placing the fish into the case. At the end of the study, fasting blood glucose levels (mmol/L) were analyzed using glucose meters (Freestyle Freedom Lite). Our methodology involved dipping test strips into cardiac blood directly after decapitation, following methods of other studies (Eames et al., 2010; Gleeson et al., 2007). Fish were fasted for 24 h prior to blood glucose testing and anesthetized before the procedure, following ethical guidelines (Gleeson et al., 2007). Anaesthetizing solution consisted of 4.2 ml of 0.4% tricaine mixed with 100 ml of circulated system water. We used three Freestyle Freedom Lite glucose meters to obtain three readings from each fish which were used to calculate the intraclass correlation coefficient (ICC). The ICC refers to correlations within a class (cluster) of data (in our case, repeated measurements of glucose readings) and is a well‐known statistical tool for measuring the reliability of an experimental method (Liljequist et al., 2019; Nakagawa & Schielzeth, 2010).

## 4 BEHAVIORAL AND STATISTICAL ANALYSIS

We analyzed all anxiety video recordings with the video tracking software Ethovision XT 14.0 (Noldus et al., 2001). In Ethovision, we created three digital zones (high, mid, and low) in the tanks for analysis. Acquisition of data began 40 s after the fish had been placed in the testing tank. This was deemed necessary as it considered the time taken to place all fish in the testing tanks and ensured the lighting and contrast had stabilized (changes occurred once researchers removed themselves from the frame).

All statistical analyses were conducted in the R environment (Version 3.4.3; R Core Team, 2021) with R Studio (Version 1.1.453; RStudio Team, 2021). We conducted two types of analyses: (1) mean and variance analyses and (2) repeatability analyses; the former involved all the traits introduced above (aversive learning, anxiety measurements, fish weight, and blood glucose levels), while the latter did not include fish weight data due to expected growth changes. Both types of analyses involved using a mixed effects model framework with sex and treatment groups (four different groups) used as fixed effects and fish ID as a random (clustering) factor in all analyses. Mixed models with the two anxiety traits required an additional scaled fixed effect (water condition, a temporal factor to control for fish being trialed in water that had not yet been changed and therefore exposed to stress hormones from other fish); as did body weight (week of measurement) following (Anwer et al., 2021).

### 4.1 Mean and variance differences

To calculate mean and variance differences in the aforementioned traits, we used linear mixed models implemented in the function *lme* in the *nlme* package (version 3.1‐148; Pinheiro et al., 2020). To model different residual variance between the four groups (i.e., heteroskedasticity), we not only specified the ‘weight’ argument in the *lme* function to do so but we also ran the same models assuming a constant variance between groups. These two models were compared by likelihood ratio tests using the *anova* function from the R ‘stats’ package (Version 3.6.2; R Core Team, 2021) to examine statistical significance for modelling different variances.

### 4.2 Repeatability

We estimated repeatability estimates of anxiety behaviors and aversive learning responses and reliability for glucose measurements, using *rptR* (Version 0.9.21), which quantified intraclass correlations, ICC (Stoffel et al., 2017); this package is based on a mixed‐effects model framework using the R package *lme4* (version 20; Bates et al., 2014). All estimates were ‘adjusted’ repeatabilities which included sex as a fixed effect (Nakagawa & Schielzeth, 2010) and were done separately for each different treatment group. For ICC of glucose readings, we included group, sex and glucose meter as fixed effects as data was not subsetted. All models incorporated fish IDs as a random effect. We obtained standard error and 95% confidence intervals (CIs) using *rptr*, which employs parametric bootstrapping (Faraway, 2016) with our models set to have 10,000 bootstrap samples. Repeatability estimates with confidence intervals not overlapping 0 were considered statistically significant.

## 5 RESULTS

### 5.1 Aversive learning assay

**FIGURE 2:**
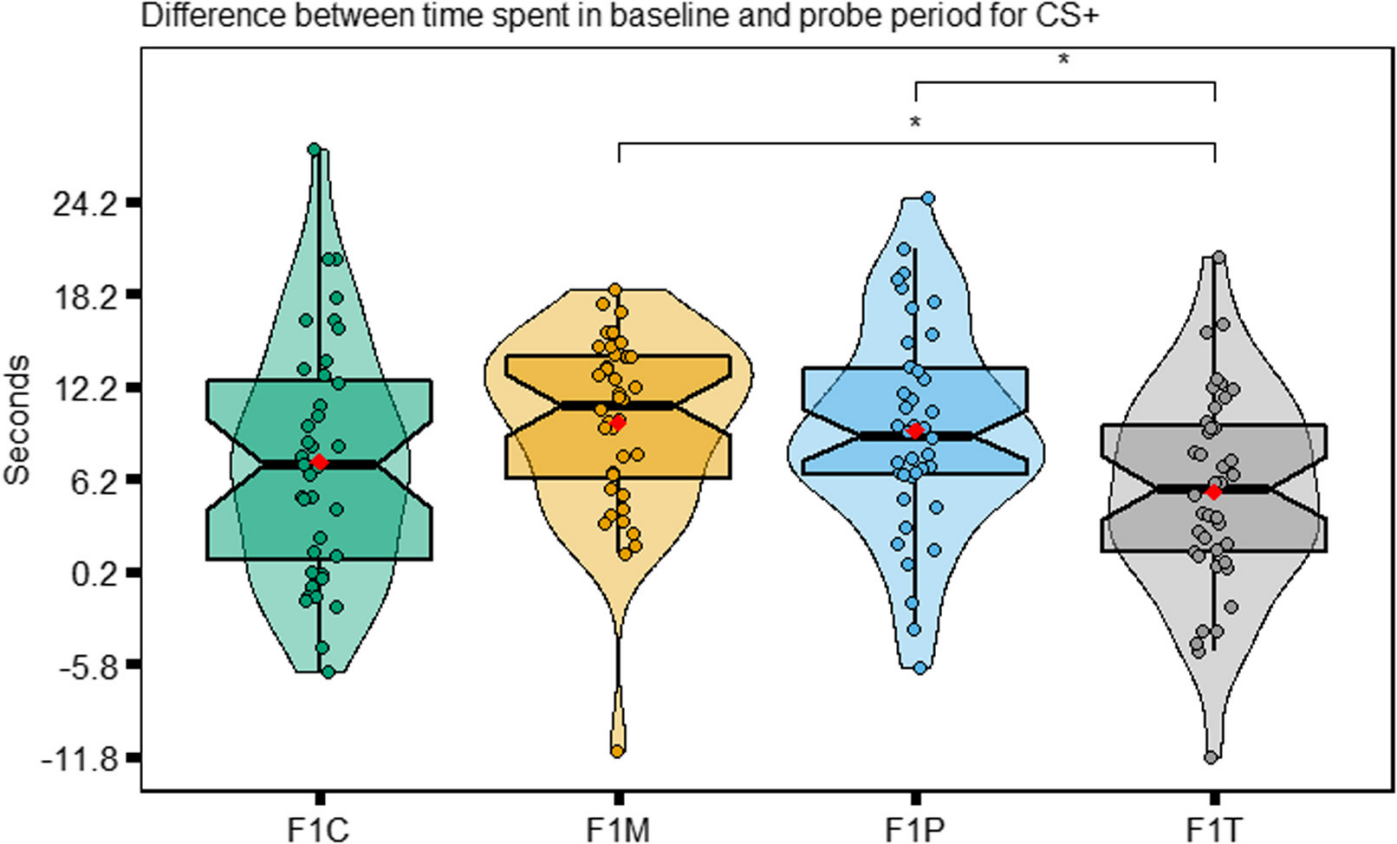
Distributions of baseline‐probe differences for each F1 zebrafish group. Each plot displays mean individual data points for males (*n* = 20 F1C, F1T, F1M; *n* = 19 F1P) and females (*n* = 20 F1C, F1T, F1M; *n* = 21 F1P) from four observations. Box plots show the median, 95% confidence interval of the median, quantiles, and outliers. Violin plots display the distribution density. Average of mean values are denoted with a red diamond. Groups without pairwise comparisons are not significantly different to one another. *Note*: *p < .05.

We quantified learning as the difference in time spent in the conditioned stimulus (CS+) before and after the aversive experience (difference = time spent in the CS+ during baseline ‐ time spent in the CS+ during probe). Overall, all differences were significantly different from 0 (see Table 1) and the F1 offspring group displayed the highest difference between the baseline and probe period (i.e., spent less time in the conditioned stimulus; *LMM F1M Intercept*, *est* = 9.57, *df* = 155, *t* = 8.08, *p* < .001; see Figure 2). There were no statistically significant differences between the F1M Group and the F1P group. However, both the F1M group and F1P group had statistically higher differences than the F1T group (*LMM F1M–F1T, est* = 4.50, *df* = 155, *t* = 3.01, *p* = .02; *LMM F1P–F1T, est* = 4.26, *df* = 155, *t* = 2.83, *p* = .03). Subsequent contrast analysis also revealed no significant differences between F1M and F1C (control group) as well as between F1P and F1C. All groups had similar variance (Figure 2) and zebrafish learning was not significantly impacted by sex.

**TABLE 1:** Intercept‐only mixed model results displaying aversion learning mean differences for F1 groups.

F1 Group	Difference	*df*	*t*	*p*‐Value
F1M	9.57	155	8.08	**<.001**
F1P	9.34	155	7.91	**<.001**
F1C	7.06	155	5.96	**<.001**
F1T	5.07	155	4.28	**<.001**

*Note*: *p*‐Values in bold indicate a significant difference from 0.

**FIGURE 3:**
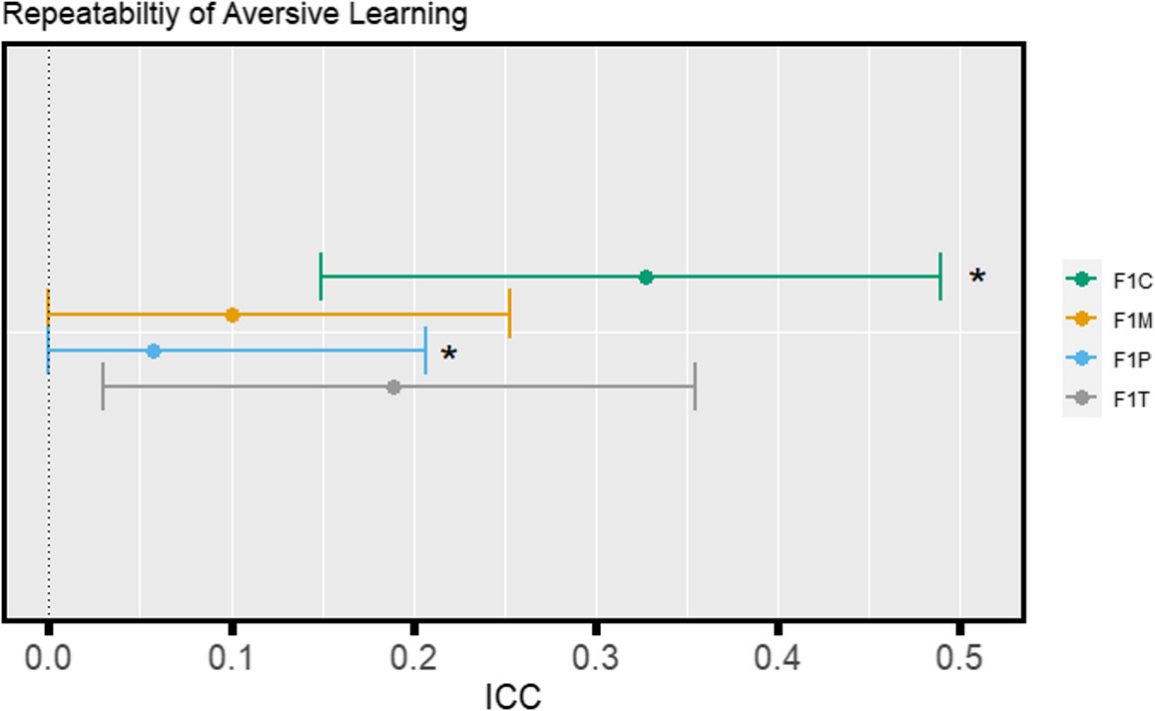
Forest plot of baseline‐probe difference repeatability estimates for each F1 group. Repeatability estimates are deemed significant if the associated 95 % confidence interval does not cross 0. Estimates with an asterisk are deemed as being statistically significantly different from one another.

Zebrafish learning was significantly (moderately) repeatable for F1 control and treatment groups (F1C: *R* = 0.33, 95% CI [0.16, 0.49]; F1T: *R* = 0.19, 95% CI [0.04, 0.36]; Figure 3) but not significantly repeatable for F1 maternal and F1 paternal groups (F1M: *R* = 0.10, 95% CI [0, 0.25]; F1P: *R* = 0.06, 95% CI [0, 0.20]; Figure 3). Furthermore, differences in repeatability estimates were statistically significant between F1C and F1P (95 % CI [0.04–0.46]; Figure 3).

### 5.2 Anxiety assay

**FIGURE 4:**
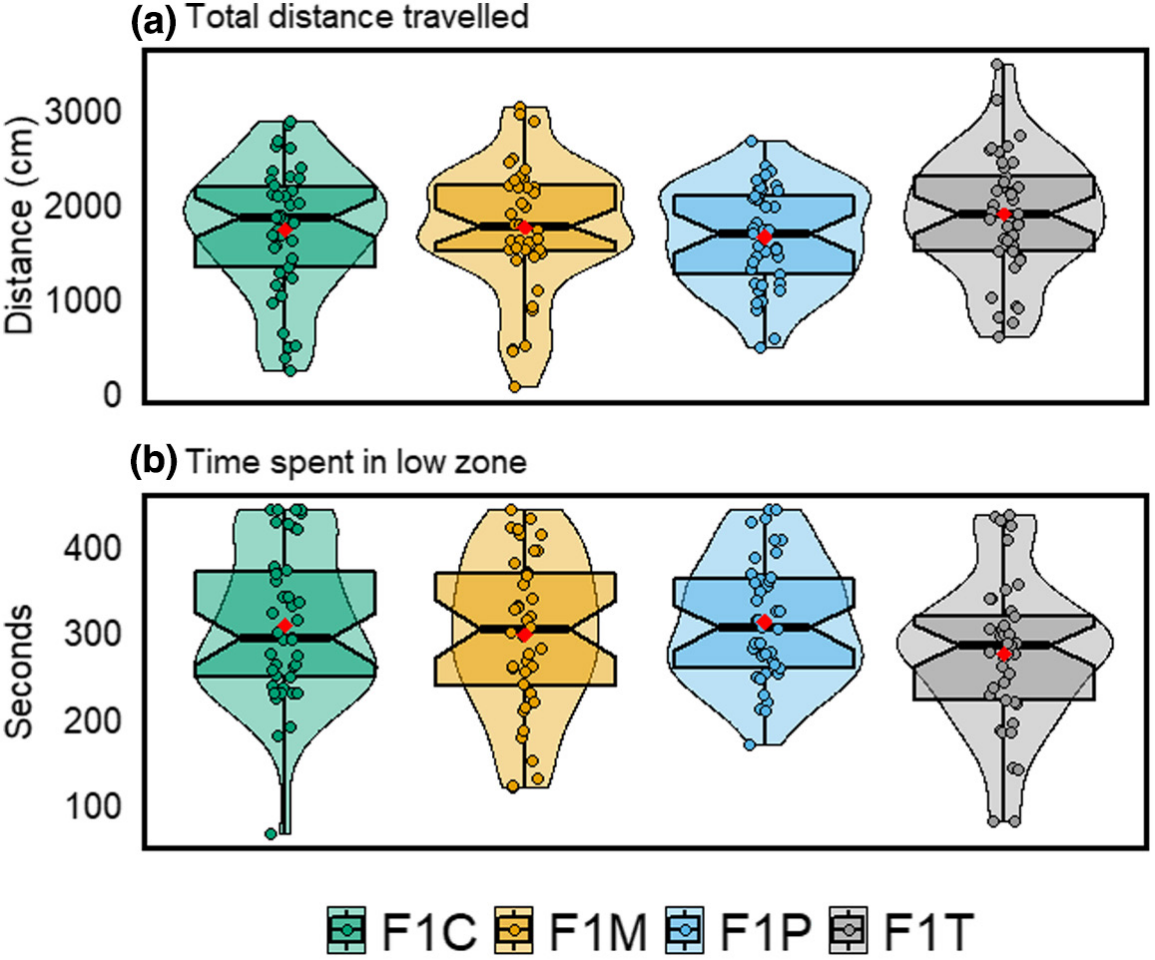
Distribution of anxiety‐associated parameters. (a) Total distance travelled (cm) and (b) time spent in the low zone (seconds) for four F1 zebrafish group. Each plot displays mean individual data points for males (*n* = 21 F1C; *n* = 20 F1T, F1M; *n* = 19 F1P) and females (*n* = 21 F1C, F1T; *n* =20 F1M; *n* = 22 F1P) from four observations. Box plots show the median, 95% confidence interval of the median, quantiles, and outliers. Violin plots display distribution density. Average of mean values are denoted with red diamonds.

**FIGURE 5:**
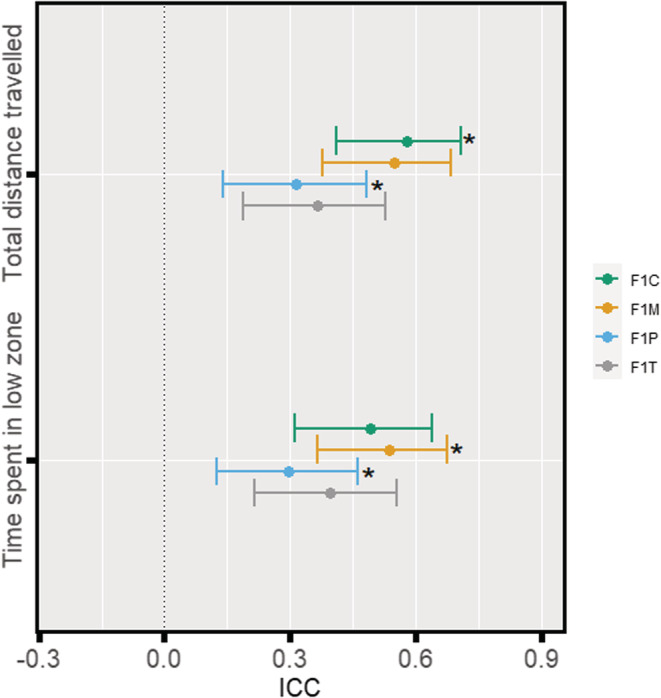
Forest plots of repeatability estimates for anxiety‐associated parameters. The plot shows: total distance travelled, and time spent in the low zone, for four F1 groups. Repeatability estimates are deemed significant if the associated 95 % confidence interval does not cross 0. Estimates with an asterisk are deemed as being significantly different from one another.

Overall, we found no statistically significant differences between F1 groups for the anxiety‐associated behaviors total distance travelled and time spent in the low zone (Figure 4 & Appendix S3: Table S1). In addition, we did not find statistically significant differences between males and females in this assay (Figure 4). As expected, as time passed after a water change, zebrafish travelled significantly less (*LMM, est* = −43.92, *df* = 476, *t* = −2.97, *p* = .003) and spent significantly more time in the low zone (*LMM, est* = 7.02, *df* = 476, *t* = 3.53, *p* < .001). Differences in variance were statistically insignificant between the four groups (Figure 4). However, the total distance travelled was significantly repeatable in all four groups (F1C: *R* = 0.58, 95% CI [0.41, 0.71]; F1T: *R* = 0.37, 95% CI [0.19, 0.53]; F1M: *R* = 0.55, 95% CI [0.38, 0.68]; F1P: *R* = 0.32, 95% CI [0.14, 0.48]; see Figure 5), as was time spent in the low zone (F1C: *R* = 0.49, 95% CI [0.31, 0.64]; F1T: *R* = 0.40, 95% CI [0.22, 0.55]; F1M: *R* = 0.54, 95% CI [0.37, 0.68]; F1P: *R* = 0.30, 95% CI [0.12, 0.46]; see Figure 5). Furthermore, differences in repeatability estimates were statistically significant between F1P and F1C (95 % CI [0.02–0.48]) for the total distance travelled, and between F1M and F1P (95 % CI [0.01–0.46]) for the time spent in the low zone (Figure 5).

### 5.3 Body weight and fasting blood glucose

Overall, male and female zebrafish from the F0 (parental) obesogenic treatment group (see our paper (Anwer, O'Dea, et al., 2022) for more details on the F0 cohort) were significantly heavier than their control counterparts after 22 weeks of diet exposure (*treatment female – control female est* = 0.13, *df* = 171, *t* = 9.28, *p* < .0001; *treatment male – control male est* = 0.06, *df* = 171, *t* = 4.32, *p* = .0002).

**FIGURE 6:**
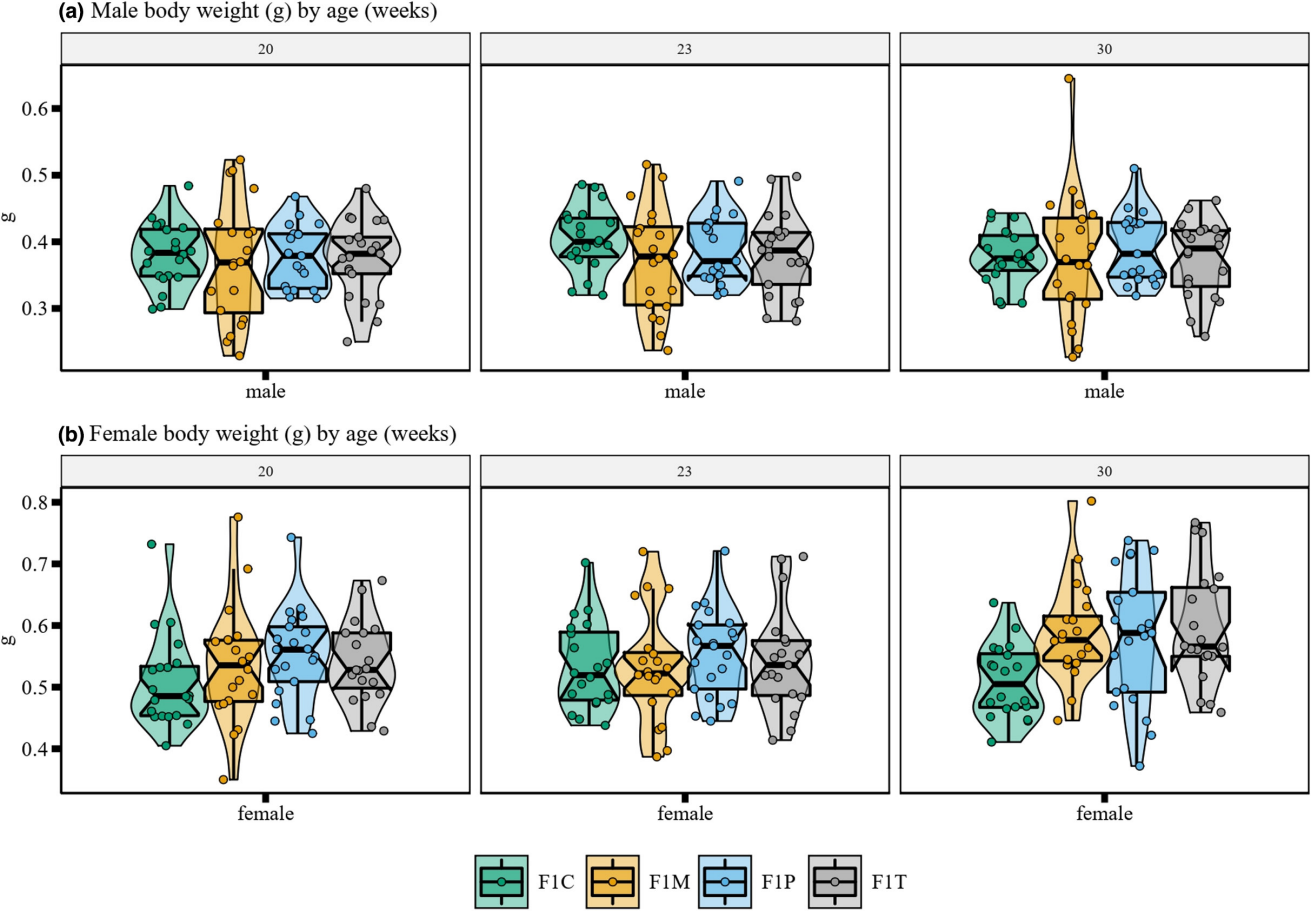
Distribution of body weight for each F1 zebrafish group. The plot shows body weights at 20, 23, and 30 weeks postfertilization subset by males (*n* = 20 F1C, F1M, F1P; *n* = 21 F1T) and females (*n* = 20 F1M, F1T; *n* = 21 F1c; *n* = 22 F1P). Box plots show the median, 95% confidence interval of the median, quantiles and outliers. Violin plots display the distribution density.

In the offspring generation, there were no statistically significant differences between groups in body weight (g) (Figure 6 & Appendix S3: Table S2). As expected, males were significantly lighter than females across all groups (*LMM*, *est* = −0.16, *df* = 313, *t* = −15.1, *p* < .001). All groups showed a slight yet significant increase in weight over weeks (*LMM*, *est* = 0.002, *df* = 313, *t* = 5.74, *p* < .001). There was no statistically significant difference in variances between the four groups.

**FIGURE 7:**
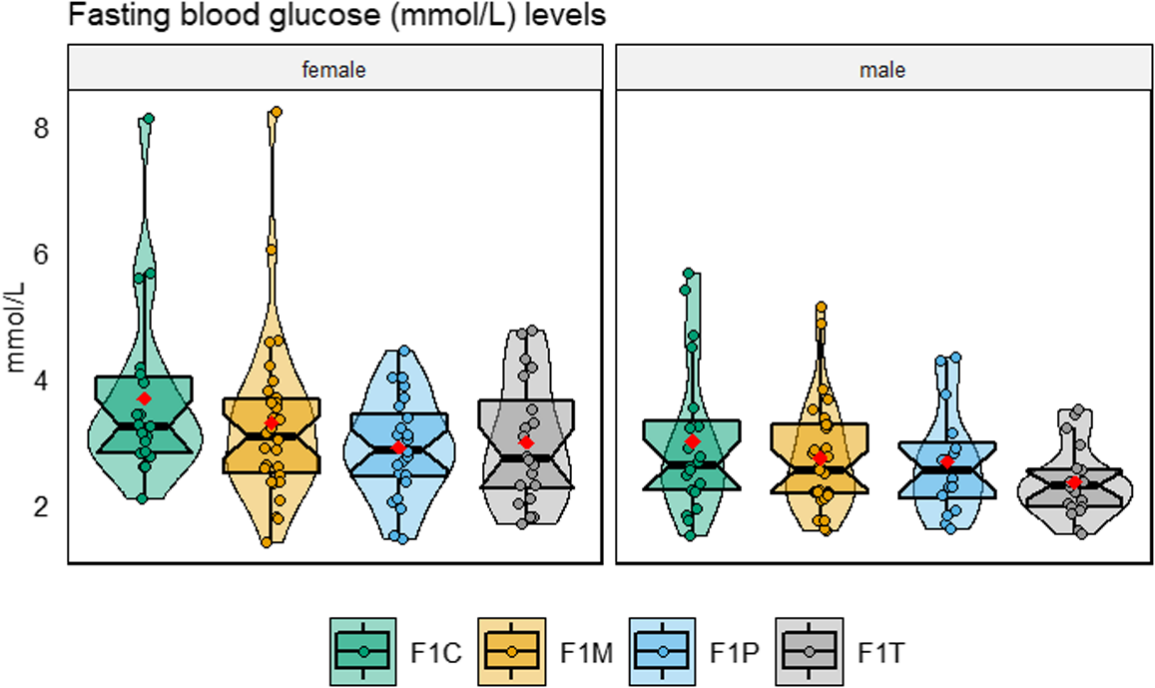
Distributions of fasting blood glucose (mmol/L) for the four F1 zebrafish groups. Plots are subset by sex. Each plot displays mean individual data points for males (*n* = 20 F1C, F1T; *n* = 29 F1M; *n* = 16 F1P) and females (*n* = 19 F1C; *n* =20 F1T; *n* = 31 F1M; *n* = 24 F1P) from three observations. Box plots show the median, 95% confidence interval of the median, quantiles, and outliers. Violin plots display the distribution density. Average of mean values are denoted with red diamonds.

Similarly, there were no statistically significant differences between groups in fasting glucose levels (mmol/L; Appendix S3: Table S3) and males had lower levels of glucose in comparison to females (*LMM*, *est* = −0.53, *df* = 173, *t* = −3.83, *p* = .001; Figure 7). However, variance levels were statistically different in each group (*p* < .001). The F1T, F1P, and F1M group had reduced variability in relation to the F1C group (by 65%, 40%, and 0.02%, respectively). Glucose measurements (obtained three times using separate glucose meters) were highly reliable (*ICC* = 0.91, 95% CI [0.89, 0.93]).

## 6 DISCUSSION

Our study aimed to address how obesogenic diets in F0 generation zebrafish could influence zebrafish cognition at F1 (intergenerational effects) in terms of both its magnitude and variability. When both parents were exposed, offspring (F1T) displayed the weakest aversive learning responses (i.e., they were deemed the least capable of learning; Figure 2). Contrary to our predictions, we found that offspring that only had one parent exposed to an obesogenic diet (F1P and F1M) displayed the strongest aversive learning responses (i.e., they were deemed the most capable of learning); and there were no significant differences in variability among the four groups (Figure 2). Zebrafish displayed no statistically significant differences in anxiety‐associated behaviors (Figure 4). Also, both males and females performed similarly in both aversive learning and anxiety assays. Furthermore, all F1 groups had similar body weights (within sex; Figure 6) and fasting blood glucose levels (Figure 7). Yet, we detected reduced variability in the glucose levels in F1 fish from the F1P and F1T groups compared to the F1C and F1M groups (Figure 6). Notably, these two groups (F1P and F1T) also showed lower repeatability in anxiety‐associated behaviors, compared to the F1C and F1M (Figure 5).

### 6.1 Intergenerational effects on aversive learning

In line with our prediction, the F1T fish displayed the weakest aversive learning responses. This is consistent with a previous study in humans, whereby if both parents were obese, their children had more difficulty with problem solving, compared to when neither parent was obese or only one parent was obese (Yeung et al., 2017). A recent study in Wistar rats also found that being born to obese parents led to a decline in spatial memory (Demir et al., 2022). Furthermore, studies in rodents by McPherson et al. (2015) and Finger et al. (2015) demonstrated that when both parents were obese, there were seemingly greater negative impacts on offspring health than either paternal or maternal obesity alone. While these two studies did not look at offspring cognition, these studies hint that there must be a combined effect occurring on offspring which would have greater adverse effects compared to when only one parent was exposed. This effect has also been seen in a human study by Fuemmeler et al. (2013) on childhood traits and weight. This study also noted that one nonobese parent may attenuate or even reverses the negative effects brought on by the obese parent suggesting some type of rescue effect. While we cannot comment on mechanisms, previous studies have alluded to epigenetics and parental inflammation as potential causative factors (Bodden et al., 2021; Hasebe et al., 2021; Mitchell et al., 2022). Interestingly, the F1C group displayed a response not significantly different from that of the F1T fish, but F1C group's response was consistent with our earlier study (i.e., zebrafish in our previous study spent a similar amount of time in the CS+ during the probe period; Mason et al., 2021). Our study in the F0 parental generation revealed that zebrafish on an obesogenic diet displayed tendencies consistent with poor cognition. That is, some individuals in the obesogenic diet group performed consistently worse in aversive learning tests than control fish, which seemed to have led to higher repeatability estimates (Anwer, O'Dea, et al., 2022). Recently, two other studies examined cognitive function in zebrafish following a HFD treatment (Meguro et al., 2019; Picolo et al., 2021). Both studies employed aversive learning assays and discovered significant impairments in memory acquisition and avoidance of aversive stimulus in obese zebrafish. While these results are important in supporting the immediate effects of HFD's on zebrafish cognition, these zebrafish studies did not extend to the F1 generation.

We found unexpected results across F1 groups, with some evidence potentially indicating adaptive parental effects that aid offspring survival (Marshall and Uller, 2007). Those F1 zebrafish that only had one parent exposed to an obesogenic diet displayed the strongest aversive learning responses, although repeatability was nonsignificant, suggesting most individuals did not perform consistently better or worse in aversive learning responses over the four trials. Our results are intriguing because others have reported that both maternal and paternal obesity could result in impaired cognitive function in offspring in rodents (Sullivan et al., 2014; Zhou et al., 2018). Despite accumulating evidence for adverse parental effects in rodent studies, not all studies concur. In a recent mouse study, male offspring born to HFD fed dams displayed no significant impairments in cognition (Zieba et al., 2019). However, Bilbo and Tsang (2010) reported offspring (F1) of parents fed a HFD performed better than their control counterparts in the Morris water maze task in rats. Similarly, in a study by Johnson et al. (2017), male offspring of females exposed to a high fat diet showed improved abilities in spatial learning and memory when compared with control male offspring. Furthermore, a meta‐analysis by Menting, Mintjens, et al. (2019a), Menting, van de Beek et al. (2019b) showed rodent offspring of obese mothers displayed similar abilities in memory tasks to control offspring. Taken together, when only one parent is exposed, there may be a mitigation effect occurring, whereby an obesogenic diet exerts a protective effect on aspects of offspring brain development (Lindsay et al., 2019). For instance, studies by Huang et al. (2015) and Rincel et al. (2016) noted offspring of prenatally stressed rats fed a HFD throughout pregnancy and lactation had improved brain development. Further studies are needed to understand the mechanisms behind such results. It is important to note, that while zebrafish in the parental generation on the obesogenic diet were significantly heavier, we did not measure physiological parameters beyond fasting blood glucose. This makes it difficult to determine whether parents in the treatment group were indeed suffering from physiological obesity, and as such, the extent to which they influenced cognition in zebrafish offspring.

### 6.2 No intergenerational effects on anxiety responses

Emotional states (i.e., mood) influence information processing and affect responses to stimuli (Nettle & Bateson, 2012). As anxiety has been shown to be closely associated with cognitive processes (Darcet et al., 2014), we also subjected F1 zebrafish to anxiety tank tests. Behavioral measurements analyzed for anxiety were significantly repeatable and displayed similar trends. The groups F1C and F1M were overall more repeatable than the groups F1P and F1T, suggesting individuals were more predictable in their behavioral profiles. While we found no significant differences between the F1 groups in anxiety‐associated behaviors, several mice studies have showcased interrelations between a perturbed emotional state and impaired cognitive performance (Ohl et al., 2003; Salomons et al., 2012). Also, in humans, heightened anxiety can elicit impaired cognitive functioning in aspects such as perception, attention, and learning (Robinson et al., 2013); and spatial and verbal working memory (Vytal et al., 2012, 2013).

### 6.3 No sex effect on aversive learning and anxiety responses

While our study found no sex differences in both anxiety and aversive learning, sex remains an important biological variable. Its inclusion in experiments has been repeatedly called for to improve the value of research, particularly in the fields of neuroscience and (bio)‐medicine (Beery & Zucker, 2011; McCarthy et al., 2017; Zajitschek et al., 2020). Sex‐specific effects are important when attempting to understand the magnitude and mechanisms of intergenerational as well as transgenerational parental effects on offspring (Hellmann, Abbas Bukhari, et al., 2020; Hellmann, Carlson, & Bell, 2020). Ignoring sex effects by pooling males and females together will result in not only underestimated effects but also the inability to question issues associated with the interrelations between intergenerational plasticity and sex‐specific selective pressures (Hellmann, Abbas Bukhari, et al., 2020). Here we demonstrated that, in zebrafish, intergenerational effects of F0 diets affect males and females in a similar manner.

### 6.4 Negligible effects on weight but variable effects on glucose in F1

All F1 zebrafish offspring displayed similar body weights, regardless of parental diet. This seems to contradict the findings that rodent studies often show offspring of mothers and fathers fed HFD's display increased weight gain as well as body fat percentage (Chambers et al., 2016; Consitt et al., 2018; Lagisz et al., 2015; Wu et al., 1998; Wu & Suzuki, 2006). A zebrafish study observed offspring of parents fed a high‐fat diet gained less weight when compared to offspring of parents fed a high‐carbohydrate diet (Türkoğlu et al., 2021). Therefore, results may vary across animal models and may also depend on the type of obesogenic diet.

Similarly, all F1 zebrafish also presented with similar fasting blood glucose levels (regardless of parental diet). Although, the control group (F1C) had highly variable levels and the F1M group had a similarly high level. In contrast, the treatment group (F1T) had the least amount of variation, followed by the F1P group, suggesting a canalization effect (Wells, 2014). A recent meta‐analysis of rodent studies discussed a similar pattern, suggesting a ceiling effect, whereby levels are at their physiological capacity, effectively reducing the amount of variation (Anwer, Morris, et al., 2022). Intriguingly, these two groups F1T and F1P were less repeatable in the anxiety assay, indicating some relationship between glucose levels and anxiety‐related behavior. Nevertheless, our results are still unexpected, as there is much evidence from rodents and zebrafish indicating impaired glucose metabolism in offspring following parental obesity (Dearden & Balthasar, 2014; do Carmo Rodrigues Virote et al., 2020; Long et al., 2010; Menting, Mintjens, et al., 2019a; Ng et al., 2010; Ribaroff et al., 2017). A few rodent studies have also shown it is not uncommon for parental HFD's to have no effect on offspring metabolic parameters (Chin et al., 2017; King et al., 2013; Platt et al., 2014). This suggests that many more factors need to be considered to determine whether offspring will experience physiological disturbances from parental obesogenic diets. As we only performed fasting blood glucose tests, it may be worthy to explore more sensitive tests in the future such as glucose tolerance tests, with previous work having shown that subtle differences can be detected in these tests despite lack of effects on fasting blood glucose (Michel et al., 2016).

### 6.5 Conclusion and future perspectives

In conclusion, our study is the first to test the intergenerational effects of an obesogenic diet on zebrafish cognition. When both parents were exposed, offspring (F1T) performed worse in aversive learning assays. However, this effect was seemingly mitigated when only one parent was exposed, resulting in stronger learning responses in the F1M and F1P groups. Repeatability estimates were also affected, with F1T offspring displaying consistently poor learning responses. While anxiety‐associated behaviors as well as fasting blood glucose were unaffected, F1P and F1T offspring had poorer repeatability for anxiety‐associated behaviors as well as less variability in glucose levels. We also found no significant influences of offspring sex. Our study examined the effects on aversive learning, but not appetitive learning. Therefore, it would be interesting to see whether appetitive learning or other memory tests (i.e., Y maze) produce similar results in zebrafish offspring. In addition, viability parameters of eggs/larvae (i.e., egg viability, mortality, and hatching) may reveal important patterns. Most notably, our study's multifactorial design allowed us to disentangle maternal and paternal effects as well as combined effects. More future studies should employ similar multifactorial experimental designs to investigate intergenerational effects on a wide range of traits not only in zebrafish but also in other animal models.

## AUTHOR CONTRIBUTIONS

**Hamza Anwer:** Conceptualization (lead); data curation (lead); formal analysis (lead); investigation (lead); methodology (lead); visualization (lead); writing – original draft (lead); writing – review and editing (lead). **Dominic Mason:** Investigation (supporting); writing – review and editing (supporting). **Susanne Zajitschek:** Supervision (lead); writing – review and editing (supporting). **Daniel Hesselson:** Resources (lead); writing – review and editing (supporting). **Daniel Noble:** Conceptualization (lead); methodology (supporting); writing – review and editing (supporting). **Margaret Morris:** Supervision (lead); writing – review and editing (supporting). **Malgorzata Lagisz:** Data curation (supporting); supervision (lead); writing – review and editing (supporting). **Shinichi Nakagawa:** Conceptualization (supporting); funding acquisition (lead); methodology (supporting); resources (lead); software (supporting); supervision (lead); writing – review and editing (supporting).

## FUNDING INFORMATION

This research was funded through an Australian Research Council Discovery grant (DP180100818) awarded to S. Nakagawa.

## CONFLICT OF INTEREST

The authors have no competing interests or conflict of interest to declare.

## Supporting information


Appendix S1

## Data Availability

All data and code can be accessed at the GitHub link: https://github.com/Apex619/Zebrafish_intergenerational_learning_obesity.
