# Supplementary material for: Intergenerational effects of overfeeding on aversive learning in zebrafish (Danio rerio)
Source: Ecol Evol. 2022 Oct 17;12(10):e9423. doi: 10.1002/ece3.9423 (PMC9596326; doi:10.1002/ece3.9423)
Supplement: Supplementary file 1 — Appendix S1 [file ECE3-12-e9423-s001.docx]

# Appendix S1

**Creating F0 experimental cohorts**

We set up 60 breeding pairs, over two days, from a stock of over 200 wildtype zebrafish. The wild-type stock was derived from of a mixture of Tübingen long fin, AB and other unidentified strains, which had been interbred for 8 – 10 generations to increase genetic diversity. We kept offspring from the 24 highest quality clutches to produce a total of 24 families. After 5 dpf, we transferred fry into independent nursery tanks upon which they were fed a standard facility diet of *Paramecium* twice daily up until 10 – 12 dpf when they were weaned onto live *Artemia* (twice a day) and dried fish food (once a day). We pseudo-randomly allocated fish following marking at 8 wpf to experimental and control tanks (4 main tanks per group each with 2 spare tanks; total of 6 tanks per group; 24 fish per tank; zebrafish were housed in 3.5 L tanks), balancing sex ratio and family representation within each tank.

**Creating F1 experimental cohorts**

We produced the F1 generation by breeding parental zebrafish after 18 weeks of dietary manipulation. Eggs were collected at 0 dpf and transferred into separate petri dishes (2 per breeding tank) containing anti-fungal egg water. Unfertilized eggs were discarded from petri dishes after a quality check at 1 dpf. After 5 dpf, 40 – 50 fry were selected randomly from each brood and transferred into separate nursery tanks (1.5 L). We established a total of 16 F1 nursery tanks (4 breeding group types with 4 replicates each – see Figure below).


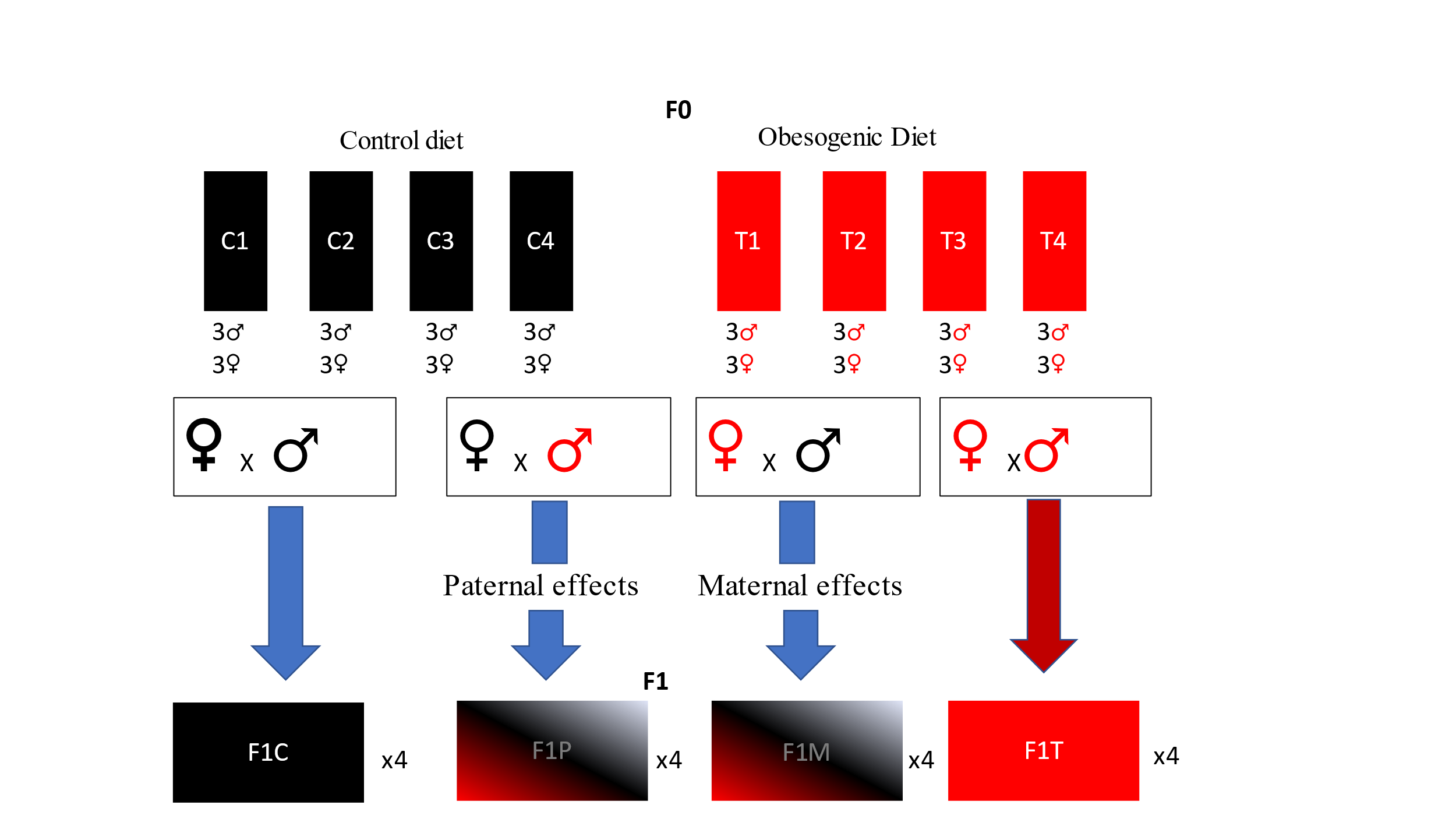


**Figure S1)** Full factorial breeding design to disentangle maternal and paternal effects of obesogenic diets. Within control (F1C) breeding involved pairing 3 males with 3 unrelated females from the same Main tank (4 Main control tanks: C1, C2, C3, C4). The same was applied for within treatment (F1T) breeding (4 Main treatment tanks: T1, T2, T3, T4). We systematically paired Main treatment tanks with Main control tanks for sex-specific breeding (T1xC1, T2xC2, T3xC3, T4xC4). Maternal groups (F1M) were bred by selecting 3 females from a given Main treatment tank and paired with 3 unrelated males from a given Main control tank. Paternal groups (F1P) were created by selecting 3 males from a given Main treatment tank and paired with 3 unrelated females from a given Main control tank.

At 30 dpf, the zebrafish fry from each nursery tank were split into two sister groups (20 – 24 fish per tank). Each sister group was transferred into a 3.5 L tank. At 60 dpf, we marked all fish from F1 tanks with Visible Implant Elastomer tags for individual identification. Following marking, fish from the sister tanks were pooled and randomly culled to 12 males and 12 females. At 90 dpf, we identified 3 tanks with the highest density (out of the 4 replicates / tanks in each F1 group type / condition) to pseudo-randomly allocate marked fish to experimental tanks (3 tanks for each F1 group type / condition, 24 fish per tank: 2 Main tanks and 1 Spare tank). We balanced sex ratio and family representation within each tank (in each condition). F1 fish were void of any dietary manipulation and fed a standard facility diet.

# Appendix S2
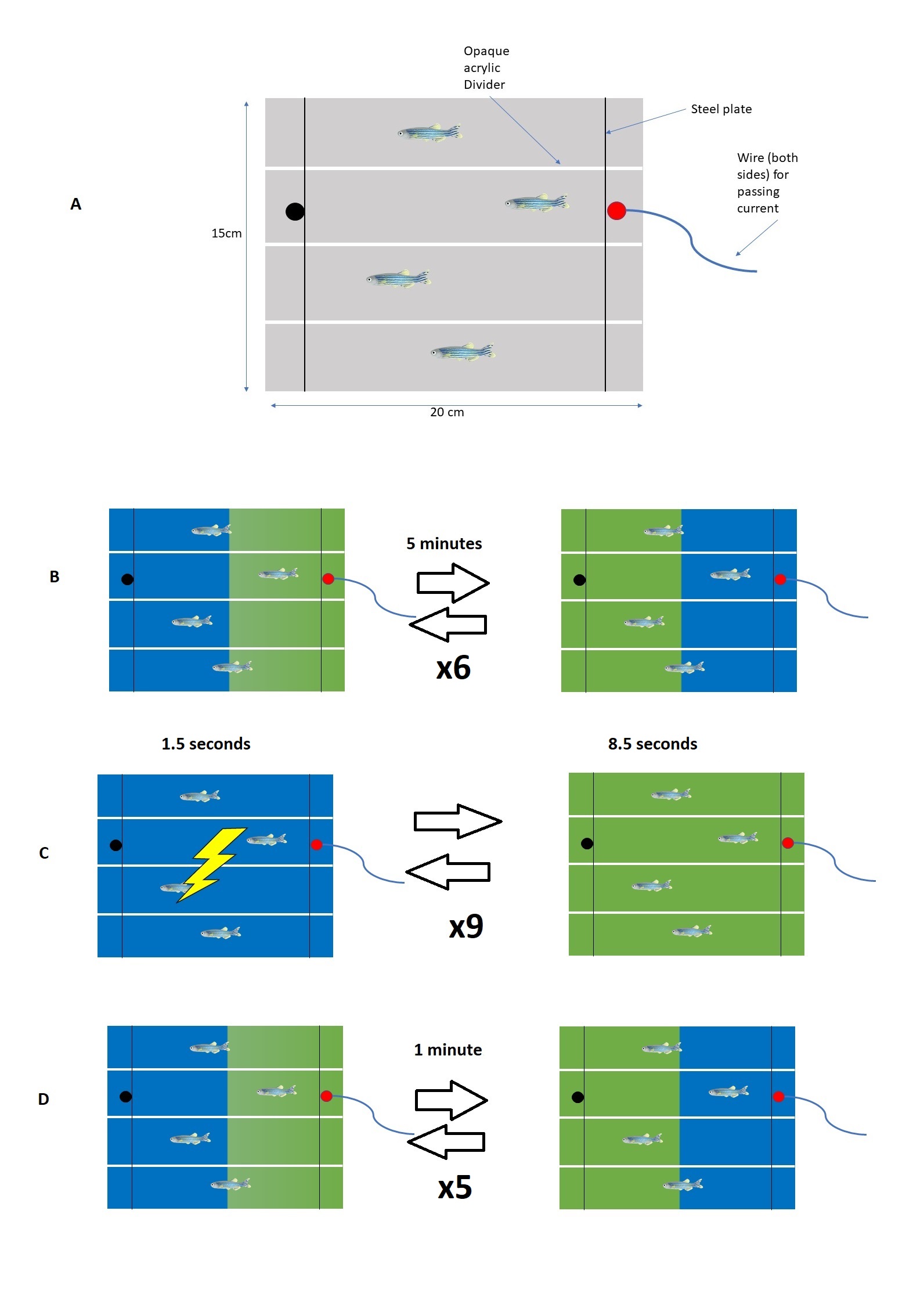


**Figure S2)** A) Aerial view of learning setup in the Zantiks tank. A blank base was displayed during the neutral acclimation period for 30 minutes B) Display during the baseline period (colours alternate sides every 5 minutes for 30 mins) C) The conditioning period; the conditioned stimuli (blue) is flashed for 1.5secs before a mild electric shock (7V, 70ms) is delivered. The base then switches to the unconditioned stimuli for 8.5 seconds D) Final stage where learning is assessed by repeating baseline protocol for 10 mins, with colours alternating sides every minute. Adapted from (Mason et al., 2021).

# Appendix S3

**Table S1*)*** Mixed model results for the anxiety parameters total distance travelled and time spent in the low zone. Contrasts are provided for group differences, sex differences and water condition. Point estimates (means) are provided as well as 95% confidence intervals (CI) and p-values. Statistically significant results are highlighted in bold.

|  | **Total distance travelled** | | | **Time spent in the low zone** | | |
| --- | --- | --- | --- | --- | --- | --- |
| *Predictors* | *Estimates* | *CI* | *p* | *Estimates* | *CI* | *p* |
| (Intercept) | 1720.85 | 1499.74 – 1941.96 | **<0.001** | 282.30 | 253.12 – 311.47 | **<0.001** |
| Tank [Paternal] | -99.29 | -378.84 – 180.26 | 0.484 | 15.76 | -21.12 – 52.65 | 0.400 |
| Tank [Within Control] | -96.19 | -378.80 – 186.43 | 0.502 | 20.77 | -16.54 – 58.07 | 0.273 |
| Tank [Within Treatment] | 75.87 | -206.80 – 358.54 | 0.597 | -12.46 | -49.77 – 24.86 | 0.511 |
| Sex [male] | 111.27 | -86.23 – 308.77 | 0.267 | 16.49 | -9.57 – 42.56 | 0.213 |
| Water condition | -43.92 | -72.99 – -14.86 | **0.003** | 7.02 | 3.11 – 10.92 | **<0.001** |

**Table S2*)*** Mixed model results for body weight of F1 zebrafish. Contrasts are provided for group differences, sex differences and week of measurement. Point estimates (means) are provided as well as 95% confidence intervals (CI) and p-values. Statistically significant results are highlighted in bold.

|  | **Weight (g)** | | |
| --- | --- | --- | --- |
| *Predictors* | *Estimates* | *CI* | *p* |
| (Intercept) | 0.54 | 0.52 – 0.57 | **<0.001** |
| Set [Paternal] | 0.01 | -0.02 – 0.04 | 0.503 |
| Set [Within Control] | -0.01 | -0.04 – 0.02 | 0.633 |
| Set [Within Treatment] | 0.01 | -0.02 – 0.04 | 0.622 |
| Sex [male] | -0.16 | -0.18 – -0.14 | **<0.001** |
| Age (Weeks) | 0.00 | 0.00 – 0.00 | **<0.001** |

**Table S3*)*** Mixed model results for fasting blood glucose levels of F1 zebrafish. Contrasts are provided for group differences and sex differences. Point estimates (means) are provided as well as 95% confidence intervals (CI) and p-values. Statistically significant results are highlighted in bold.

|  | **Fasting blood glucose** | | |
| --- | --- | --- | --- |
| *Predictors* | *Estimates* | *CI* | *p* |
| (Intercept) | 3.28 | 2.97 – 3.59 | **<0.001** |
| Group [Paternal] | -0.26 | -0.68 – 0.16 | 0.225 |
| Group [Within Control] | 0.32 | -0.10 – 0.75 | 0.138 |
| Group [Within Treatment] | -0.35 | -0.77 – 0.08 | 0.106 |
| Sex [male] | -0.53 | -0.84 – -0.22 | **0.001** |
